# Effects of oxygen functional groups and FeCl_3_ on the evolution of physico-chemical structure in activated carbon obtained from Jixi bituminous coal†

**DOI:** 10.1039/c7ra12928a

**Published:** 2018-02-26

**Authors:** Dongdong Liu, Boyin Jia, Xiujuan Liu, Bojun Zhao, Jihui Gao, Qingxi Cao, Shaohua Wu, Yukun Qin

**Affiliations:** College of Engineering and Technology, Jilin Agricultural University Changchun 130118 China; College of Animal Science and Technology, Jilin Agricultural University Changchun 130118 China jiaboyin@139.com; Petrochina Daqing Petrochemical Company Daqing 163000 China; School of Energy Science and Engineering, Harbin Institute of Technology Harbin 150001 China gaojihui0809@163.com

## Abstract

It is crucial to increase the values of *S*_BET_/burn-off ratio to achieve activated carbon (AC) with a higher SO_2_ adsorption capacity at a low cost from flue gas. In this study, at first, Jixi bituminous coal was used as a raw material to prepare a series of pre-treated samples by oxidation treatment and adding different amounts of the FeCl_3_ catalyst. Then, the AC samples were prepared by pyrolysis under a N_2_ atmosphere and physical activation with CO_2_. Finally, the change in the physico-chemical structure of different samples was determined to study the effects of oxygen functional groups and FeCl_3_. The results show that the rapid growth of mesopores is mainly influenced by the evolution of oxygen functional groups, whereas the micropores are mainly influenced by the FeCl_3_ catalyst during pyrolysis. These effects can also further improve the size and the carbon type of the aromatic structure from a different perspective to promote the disordered microstructure of treated chars (1FeJXO15-800H, 3FeJXO15-800H and 6FeJXO15-800H) as compared to the ordered microstructure and less pores of the un-pretreated char (JX-800). Then, the active sites can no longer be consumed preferentially in the presence of the catalyst; this results in the continuous disordered conversion of the microstructure as compared to the ordered conversion of JX-800 char during activation. On the one hand, the developed initial pores of 1FeJXO15-800H, 3FeJXO15-800H, and 6FeJXO15-800H chars promote the favorable diffusion of activated gas, following the non-hierarchical development. On the other hand, the presence of Fe-based catalysts facilitates the etching of carbon structure and the rapid and continuous development of the micropores, hindering the severe carbon losses on the particle surface. Finally, the 3FeJXO15-800H char with a high value of *S*_BET_ (1274.64 m^2^ g^−1^) at a low burn-off value (22.5%) has the highest *S*_BET_/burn-off ratio value of 56.65 m^2^ g^−1^/%, whereas the JX-800 char with a low value of *S*_BET_ (564.19 m^2^ g^−1^) at a burn-off value of 58.2% has the lowest *S*_BET_/burn-off ratio value of 9.69 m^2^ g^−1^/%. Therefore, the presence of oxygen functional groups and FeCl_3_ has obviously changed the evolution of the physico-chemical structure in activated carbon to effectively enhance the values of *S*_BET_/burn-off.

## Abstract 2

Effects of oxygen functional groups and FeCl_3_ on evolution of physico-chemical structure in activated carbon to increase its value *S*_BET_/burn-off.

## Graphical abstract

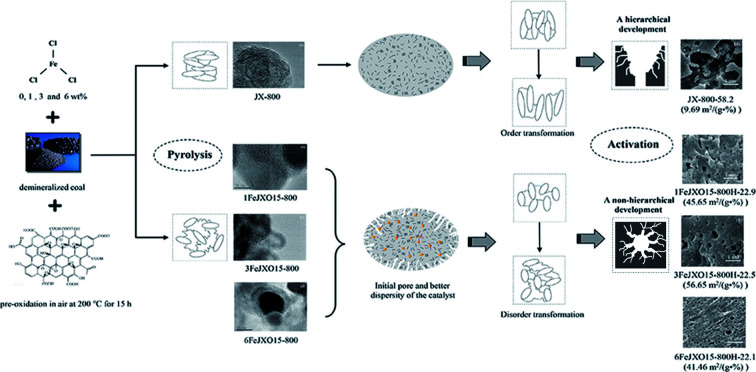

## 1 Introduction

At present, biomass is mainly used as a raw material for the preparation of activated carbon (AC). Because of the limited sources of raw materials, the production of biomass-based AC is relatively low and its price is relatively high. However, anthropogenic SO_2_ emission from large coal-fired power plants has been considered as a major gas pollutant for a long time. Traditional biomass-based AC is unable to satisfy the desulfurization requirements in coal-fired power plants; therefore, AC can be produced by coal as the most suitable raw material instead of traditional biomasses and some wastes and the traditional physical activation with steam or CO_2_ resulting from high-temperature flue gas. A higher specific surface area (*S*_BET_) is conducive for desulfurization in the existence of a hierarchical structure (micro- and mesopores).^1–4^ At present, industrial application of coal-based AC has been hindered owing to the relatively low desulfurization property and the relatively high cost, and these are associated with more carbon loss on the particle surface that cannot promote the number of pores during activation; this results in lower values of the *S*_BET_/burn-off ratio. In previous years, coal-based AC with the *S*_BET_ values between 500 and 800 m^2^ g^−1^ at the high burn-off values of approximately around 40–60% during activation has been produced by most of the researchers using physical activation.^5–7^ Substantially, higher values (200–1100 m^2^ g^−1^) have been reported by San Miguel *et al.*^8^ for activation with steam and CO_2_ at 925–1100 °C, but with burn-off levels up to 80%. These results show that it is necessary to operate at high burn-off to reach a higher *S*_BET_*via* physical activation. However, it is difficult to obtain coal-based AC with a higher *S*_BET_ at low burn-offs during activation only by adjusting the activation conditions (such as temperature and type and dosage of the activating gases).^9,10^ The specific analysis is as follows: the number of initial pores of precursors produced by pyrolysis and the conversion tendency of the carbon structure of precursors during activation have important effects on the ideal AC production. At first, in the beginning of the activation process, a small amount of initial pores can hinder the diffusion of the activated gas into the particles' interior to produce more pores; this leads to the occurrence of more reactions on the particle surfaces.^11–14^ Then, with an increase in the activation time, the highly ordered conversion of the carbon structure at high temperatures can reduce the number of active sites; this hinders the sustained growth of micropores inside the particles and continually increases the external loss of quality.^15^ Therefore, the simultaneous resolution of the abovementioned two problems is the key to achieve a high *S*_BET_/burn-off value.

Most of the studies reported in literature suggest that the simultaneous introduction of oxygen functional groups produced by air pre-oxidation and metal catalysts may solve the abovementioned problems. At first, more pores in precursors can be produced by the evolution of oxygen functional groups during pyrolysis.^5,6,16^ Francisco *et al.*^17–21^ showed that the evolution of oxygen functional groups changed the pore development, and the mesopores were first produced followed by the formation of micropores with an increase in the cyclic oxygen chemisorption–desorption number. In a former study,^22^ we reported that more active sites and a porous structure of oxidized chars were created by the evolution of oxygen-containing structures with different stabilities; this led to the rapid diffusion of the activated gas into the particles' interior during activation. However, the rapid consumption of oxygen-containing active sites and ordered conversion of carbon structure occurred with an increase in the activation time; this led to high burn-off. Then, the existence of metal catalysts may improve the conversion of the carbon structure that promotes gasification efficiency. The catalytic mechanism of gasification has been investigated by some researchers.^23–26^ They believed that some intermediates (such as C(O) and M–C–O) were formed first in the presence of catalysts, and then, these intermediates acted as active sites that could react with a gasifying agent to increase the gasification rate. In addition, in our previous study,^27^ we found that FeCl_3_ at different amounts could improve the decomposition and condensation of the microstructures during pyrolysis in various degrees. However, the rapid polymerization of Fe-based catalysis led to severe deactivation of catalysts, and there were not enough initial pores in the Fe-based precursor to ensure gas diffusion during activation; this led to a relatively low *S*_BET_/burn-off ratio value, which was in a good agreement with other studies.^28,29^ Therefore, the effects of each oxygen functional group and FeCl_3_ catalysts have been independently analyzed in our previous study. However, the effects of oxygen functional groups and Fe-based catalyst on the evolution of the physico-chemical structure in the process of formation of AC have rarely been studied in detail.

In this study, a series of coal samples were prepared by pre-oxidation in air at 200 °C for 15 h and loading various amounts of the FeCl_3_ catalyst (1 wt%, 3 wt%, and 6 wt%) as a cheap metal catalyst into the coal. The effects of oxygen functional groups and FeCl_3_ catalyst on the physical and chemical structure of coal char in the whole preparation process were investigated. The feature parameters of all the samples were obtained by transmission electron microscopy (TEM), scanning electron microscopy (SEM), nitrogen adsorption, X-ray diffraction (XRD), and Raman spectroscopy.

## 2 Experimental

### 2.1. Sample preparation

Jixi bituminous coal from China was used in this experiment, which was crushed and sieved to a particle size of 250–380 μm. To eliminate the interference of ash in coal, the sample coal was processed using 6 mol L^−1^ HCl and 40 wt% HF sequentially in accordance with the demineralization procedure.^30^ After the demineralization procedure, the proximate and ultimate analysis data of the acid-treated samples were obtained, as shown in Table 1. The ash content in the acid-treated sample was below 1%, and this sample was denoted as JX. Then, JX was oxidized in air at 200 °C for 15 h and was denoted as JXO15; moreover, a predetermined amount of FeCl_3_ powder (0.03 g, 0.09 g, and 0.18 g) and 3 g of oxidized coal (JXO15) were added to an aqueous solution. The resulting mixture was stirred for 24 h in a sealed beaker at ambient temperature, evaporated under vacuum, and dried at 100 °C overnight before the pyrolysis experiment; these samples were denoted as 1FeJXO15, 3FeJXO15, and 6FeJXO15.

**Table 1:** Proximate and ultimate analyses of the acid-treated sample

Sample	Proximate analysis (wt%)				Ultimate analysis (wt_daf_%)				
	V_ad_	FC_ad_	A_ad_	M_ad_	C_daf_	H_daf_	O_daf_^a^	N_daf_	S_daf_
JX	39.66	56.60	0.12	3.62	74.81	19.49	4.01	1.31	0.38

a By difference; ad (air-dried basis): the coal in dry air was used as a benchmark; daf (dry ash free basis): the remaining component after the removal of water and ash in coal was used as a benchmark.

### 2.2. Experimental process

The activated carbon was prepared by a conventional fixed bed reactor, and the more detailed process is as follows: (1) pyrolysis experiment: 3 g of the sample coals (JX, 1FeJXO15, 3FeJXO15, and 6FeJXO15) were placed in a three-stage fixed-bed reactor that was then heated at 8 °C min^−1^ to 800 °C and then maintained for 60 min in a N_2_ flow of 450 mL min^−1^. Next, the sample coals were rapidly cooled under a N_2_ atmosphere and denoted as JX-800, 1FeJXO15-800, 3FeJXO15-800, and 6FeJXO15-800. To eliminate the interference of Fe-based compounds in chars for the results of XRD and Raman, some char samples including Fe-based compounds were treated with 0.2 mol L^−1^ HCl and washed with distilled water to remove chloride ions; these samples were denoted as 1FeJXO15-800H, 3FeJXO15-800H, and 6FeJXO15-800H. Fig. S1† shows the XRD phase analysis results of 6FeJXO15-800 and 6FeJXO15-800H. (2) Activation experiment: these typical chars were continuously heated up to 900 °C under a N_2_ atmosphere and then held for appropriate times under a CO_2_ flow of 450 mL min^−1^ to obtain AC with different porous structures at different burn-offs. After activation, the CO_2_ flow was replaced with N_2_ to cool them down to room temperature. In addition, the char samples still included Fe-based compounds, which could interfere with the measurements and curve-fitted results of XRD and Raman owing to their fluorescence effects.^31,32^ In order to obtain accurate results, some AC samples including Fe-based compounds was treated using 0.2 mol L^–1^ HCl, and these treated samples were denoted as JX-800, 1FeJXO15-800H, 3FeJXO15-800H, and 6FeJXO15-800H-burn-off values.

### 2.3. Measurement analysis

The transmission electron microscope (TEM, Tecnai G2 F30) was operated at 200 kV to visualize the structural changes. SEM (Quanta 200) was performed using an instrument operated at 200 kV to visualize the surface topography of samples. Structural parameters of the crystalline structures were obtained by the D/max-RB X-ray diffractometer, with the measurements performed in the 2*θ* range from 10° to 80° at a scan rate of 3° min^−1^. Next, the char powders were investigated by Raman spectroscopy using a 532 nm wavelength laser and observing the scattering in the range of 1000–1800 cm^−1^. The pore structure characteristics of the prepared samples were obtained by nitrogen adsorption at the analysis temperature of 77 K using a Micromeritics adsorption apparatus (ASAP2020), with the measurements performed for the adsorption data in the relative pressure (*P*/*P*_0_) range from 10^−7^ to 1.^33^ The prepared samples were degassed under vacuum at 473 K for 12 h before N_2_ adsorption analysis. The specific surface area (*S*_BET_), the micropore area (*S*_mic_), the micropore volume (*V*_mic_), and pore size distribution were calculated using the Brunauer–Emmett–Teller (BET) equation, *t*-plot method, Horvath-Kawazoe (HK) method, and the nonlocal density functional theory (NLDFT), respectively.^34–36^ In addition, the total pore volume (*V*_t_) was determined at the 0.98 relative pressure.

## 3 Results & discussion

### 3.1. TEM analysis of coal chars produced by pyrolysis

**Fig. 1:**
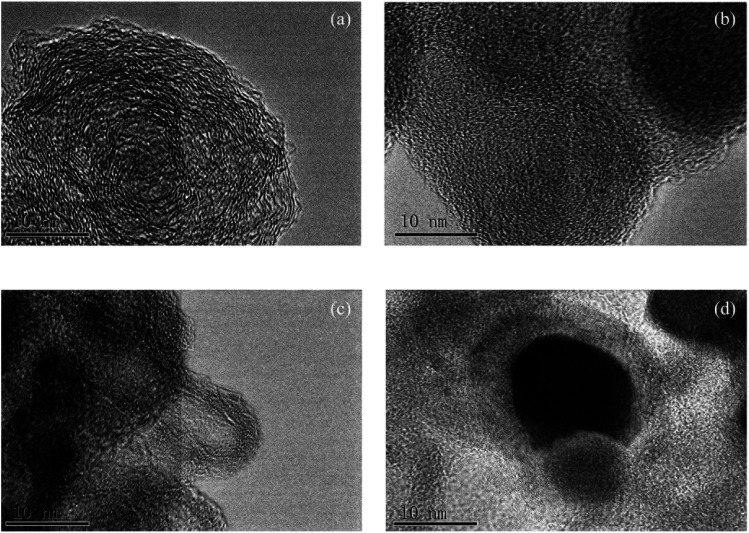
TEM images of coal chars with different amounts of FeCl_3_ (a) JX-800; (b) 1FeJXO15-800; (c) 3FeJXO15-800; and (d) 6FeJXO15-800.

Several TEM images of different coal chars at a high resolution are shown in Fig. 1. A large amount of ordered crystalline layers could be found near a small quantity of amorphous carbon for JX-800, as shown in Fig. 1(a). Then, as the amount of FeCl_3_ additive increased from 1 wt% to 3 wt%, there were more interlaced arrangements between amorphous carbon and crystallite layers and a lot of amorphous regions for 1FeJXO15-800 and 3FeJXO15-800, as shown in Fig. 1(b) and (c), indicating that the degree of order in microstructure decreased obviously. However, sphere-like particles encapsulated by multi-layer amorphous carbons can be found near some long, parallel-aligned crystallite layers having different orientations for 6FeJXO15-800, as shown in Fig. 1(d), and this phenomenon is related to the mechanism of metal-catalyzed graphitization.^37–39^ Compared to the literature^27^ regarding the TEM image (unoxidized coal chars with 6 wt% FeCl_3_), closely arranged crystalline layers of 6FeJXO15-800 were broken, and blurred boundaries of crystalline layers, smaller diameter, and more amorphous regions were also observed in Fig. 1(d). The abovementioned results show that the existence of oxygen functional groups can hinder the concentration and amalgamation of the FeCl_3_ catalyst at high temperatures; thus, the effects of oxygen functional groups and FeCl_3_ catalyst can promote the obvious disordered arrangement of the microstructure during pyrolysis.

### 3.2. Pore structure analysis of coal chars produced by pyrolysis

**Fig. 2:**
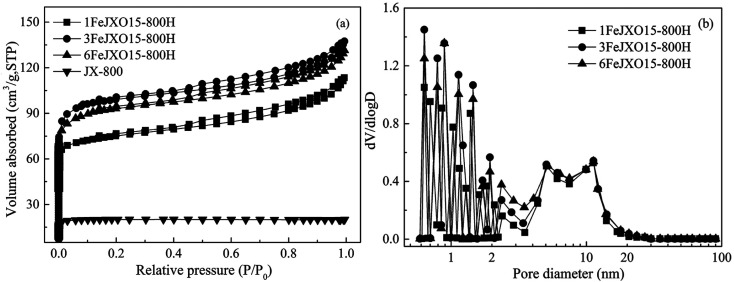
N_2_ adsorption isotherms and pore-size distributions of different coal chars obtained at 800 °C.

The N_2_ adsorption isotherms and the pore-size distribution of different coal chars are shown in Fig. 2. At first, the N_2_ adsorption capacities of JX-800 were very small, indicating fewer pores. This result is related to the formation of metaplast materials that block the pores during pyrolysis. Then, the N_2_ adsorption isotherms of 1FeJXO15-800H, 3FeJXO15-800H, and 6FeJXO15-800H were related to a type I at low pressures and type IV at high pressures according to the IUPAC classification. These isotherms began to branch, and a hysteresis loop could be observed as the relative pressure continued to increase; this indicated the formation of mesopores. With an increase in the amount of FeCl_3_ additive from 1 wt% to 3 wt%, the N_2_ adsorption capacities at low pressures increased evidently and their knee became broader; however, 6FeJXO15-800H exhibited an obvious decrease at low pressures; then, there were no obvious changes in their isotherms at high pressures. Alternatively, the pore-size distributions of different coal chars were relatively broad, where the size range was 0.6–20 nm, as observed from Fig. 2(b). As the amount of FeCl_3_ addition increased from 1 wt% to 3 wt%, the volume of the micropores obviously increased; this indicated an increase in the micropore quantity and the presence of wider micropores. These changes might be related to catalytic cracking characteristics of FeCl_3_. Gong *et al.*^30^ proved that the cleavage of the microcrystalline structure was occurred by adding Fe-based catalysts to release more volatile matter (such as CO or CO_2_); this led to formation of pores. However, the micropore volume in 6FeJXO15-800H exhibited an obvious decrease, which was related to the concentration and amalgamation of FeCl_3_ catalyst at high temperatures. In addition, the size distribution of their mesopores was similar, but an increase in mesopore volume from 3 to 5 nm for 6FeJXO15-800H was observed by removing the nanoparticles occupying the inner space of coal chars. These results indicated that the evolution of oxygen functional groups mainly facilitated the development of mesopores, and the FeCl_3_ catalyst could efficaciously promote the formation of micropores during pyrolysis. Therefore, the effects of oxygen functional groups and FeCl_3_ catalyst can simultaneously promote the development of mesopores and micropores of coal chars during pyrolysis.

### 3.3. Crystal structure analysis of coal chars produced by pyrolysis

**Fig. 3:**
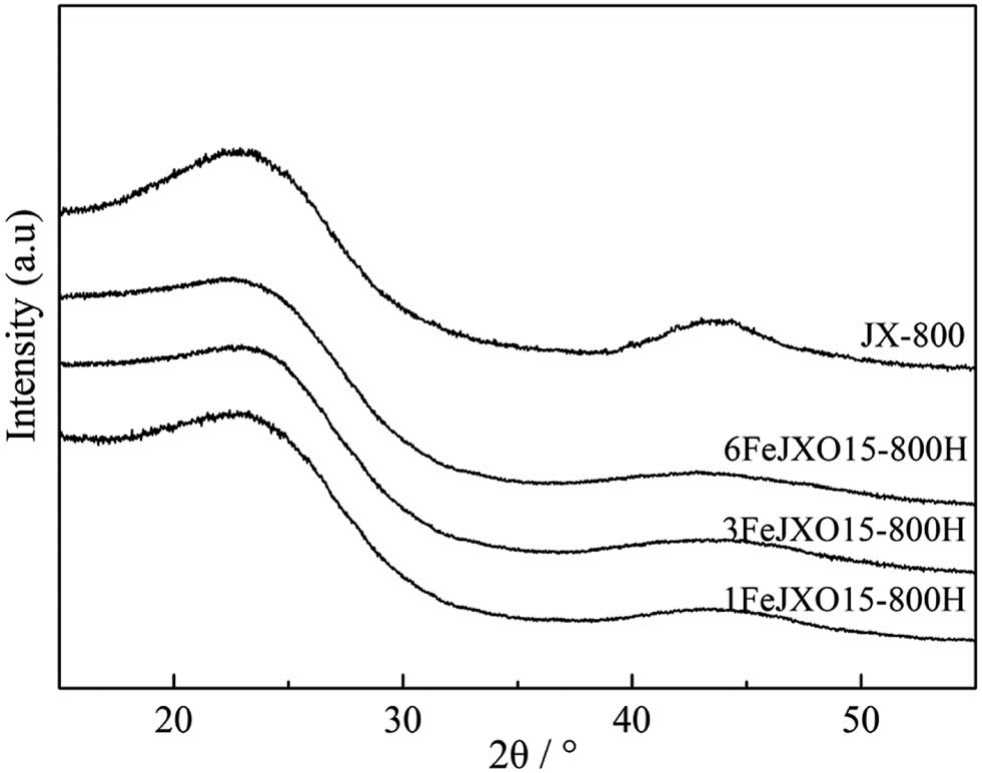
XRD profiles of different coal chars obtained at 800 °C.

The XRD profiles of different coal chars are given in Fig. 3. There were two obvious broad diffraction peaks at 2*θ* = 24°–27° and 41°–44° in all the samples, which were related to an interplanar spacing between two aromatic layers and the degree of condensation of aromatic layers.^40^ According to the peak fitting treatment used by Liu *et al.*,^27^ the XRD profiles of all the samples were processed to obtain some important parameters (such as the aromatic structure layer distance *d*_002_, stacking height *L*_c_, width *L*_a_, and the number of aromatic layers *N* = *L*_c_/*d*_002_) regarding the microcrystalline structure, as shown in Table 2.

**Table 2:** XRD data of different coal chars obtained at 800 °C

	JX-800	1FeJXO15-800H	3FeJXO15-800H	6FeJXO15-800H
*L*_a_ (Å)	24.91	25.71	25.55	25.13
*L*_c_ (Å)	13.72	12.95	12.86	12.43
*d*_002_ (Å)	3.50	3.69	3.72	3.76
*N*	3.77	3.51	3.50	3.31

The 002 and 100 peaks observed for JX-800 were most obvious, the *d*_002_ value reached a minimum of 3.50 Å, and the *L*_c_ value reached a maximum of 13.72 Å as compared to that of other samples. The microcrystalline structure of JX-800 was transformed into a highly ordered graphite-like structure because of the presence of a plastic behavior. The appearance of the metaplast material facilitated the movement and orientation adjustment of aromatic layers during pyrolysis; this resulted in the rapid stacking and condensation of aromatic layers.^5,6,11–14^ Then, the *L*_a_, *L*_c_, and *N* values of 1FeJXO15-800H, 3FeJXO15-800H, and 6FeJXO15-800H decreased, and the *d*_002_ values increased; this indicated that the microcrystalline structure was transformed into a type of disordered structure during pyrolysis in the presence of FeCl_3_ catalyst and oxygen functional groups. Compared to that of the oxidized char (JXO15-800) without the addition of the FeCl_3_ catalyst reported in our previous study,^22^ the degree of disorder in 1FeJXO15-800H, 3FeJXO15-800H, and 6FeJXO15-800H was further promoted by increasing the amount of FeCl_3_ catalyst, and Fe might penetrate into an aromatic structure during pyrolysis to enlarge the aromatic structure layer distance (*d*_002_). Alternatively, the decrease in the *L*_a_ and *L*_c_ values was related to the catalytic cracking characteristics of the FeCl_3_ catalyst during pyrolysis. Murakami and William *et al.*^41,42^ found that Fe-based compounds promoted the decomposition of aromatic structure to form more free radicals at the beginning of pyrolysis and then also hindered the further polymerization of free radicals to form a disordered microstructure at a later stage of pyrolysis. The abovementioned results indicated that the effects of oxygen functional groups and FeCl_3_ catalyst can further simultaneously improve the size of the microcrystalline structure from a different perspective to promote the degree of disorder in aromatic structure during pyrolysis; however, the catalytic cracking characteristics of the FeCl_3_ catalyst might play a more important role in hindering the vertical stacking and condensation of aromatic layers during pyrolysis.

### 3.4. Carbon structure analysis of coal chars produced by pyrolysis

**Fig. 4:**
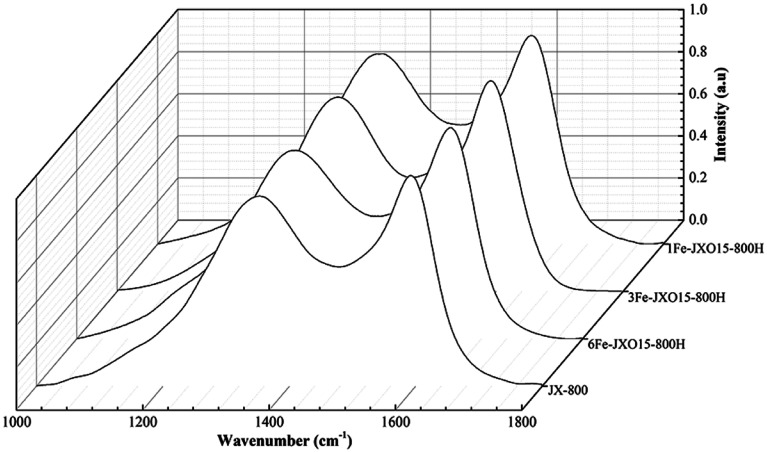
Raman spectra of different coal chars obtained at 800 °C.

The Raman spectra of the different coal chars are shown in Fig. 4. The assignment of the five bands was carried out according to the peak fitting treatment using five bands reported by Li *et al.*^43^ The Raman spectra of all the samples were processed with a smoothing function, baseline correction, and normalization using the Origin 9.1 software to obtain the parameters of different hybrid carbon structures. Importantly, the different band area ratios represent relative structure,^44,45^ and this is ascribed to the following: (1) *A*_D1_/*A*_G_ can be related to defect degree of the microcrystalline structure; (2) *A*_D3_/*A*_G_ is considered as amorphous carbon; (3) *A*_D1_/*A*_D3_ can indicate the ratio between big rings relative to small fused rings in chars; and (4) *A*_D4_/*A*_G_ can be described as the relative quantity of cross-linking bonds. In particular, the defect (*A*_D1_/*A*_G_) and cross-linking bonds (*A*_D4_/*A*_G_) can also be related to active sites in the samples. The parameters of carbon structure of carbonized chars are given in Table 3.

**Table 3:** Raman data of different coal chars obtained at 800 °C

	JX-800	1FeJXO15-800H	3FeJXO15-800H	6FeJXO15-800H
*A*_D1_/*A*_G_	3.197	3.770	3.877	3.987
*A*_D3_/*A*_G_	1.922	2.715	2.820	2.955
*A*_D4_/*A*_G_	0.611	0.849	0.885	0.936
*A*_D1_/*A*_D3_	1.663	1.388	1.375	1.349

The narrow full width at half maximum of the D peak and G peak were observed for JX-800, as shown in Fig. 4. The *A*_D1_/*A*_G_, *A*_D3_/*A*_G_, and *A*_D4_/*A*_G_ values of JX-800 reached minima, whereas the *A*_D1_/*A*_D3_ value of JX-800 reached a maximum as compared to that of other samples; this indicated the presence of an ordered material with lower reactivity. Then, the *A*_D1_/*A*_G_, *A*_D3_/*A*_G_, and *A*_D4_/*A*_G_ values of 1FeJXO15-800H, 3FeJXO15-800H, and 6FeJXO15-800H increased, whereas the *A*_D1_/*A*_D3_ values decreased. These changes in hybrid carbon structures led to an increase in reactivity during pyrolysis. Compared to the oxidized char (JXO15-800) without the addition of FeCl_3_ catalyst reported in our previous study,^22^ the degree of change in 1FeJXO15-800H, 3FeJXO15-800H, and 6FeJXO15-800H was promoted further by increasing the amount of FeCl_3_ catalyst; this presented more disordered conversion of the carbon structure during pyrolysis. These results indicated that Fe-based components hindered the transformation of the isolated and defective sp^2^ structure (D_1_ peak) into the crystalline sp^2^ structure (G peak) and promoted the splitting of big aromatic rings (D_1_ peak) to form the amorphous sp^2^ bonding carbon atoms (D_3_ peak); this led to a decrease in *A*_D1_/*A*_D3_. In addition, the metal components (M) might link with the carbon matrix and oxygen functional groups to form a C–O–M bond during pyrolysis; this would result in an increase in the sp^2^–sp^3^ bonding carbon atoms (D_4_ peak).^41^ These results indicate that the effects of oxygen functional groups and FeCl_3_ catalyst can further improve the conversion of the carbon structure during pyrolysis to promote the number of active sites including the defects, the cross-linking bonds, and small aromatic rings, and the existence of oxygen functional groups can further promote the catalytic characteristics of the FeCl_3_ catalyst during pyrolysis.

### 3.5. Crystal structure analysis of typical chars during activation

**Fig. 5:**
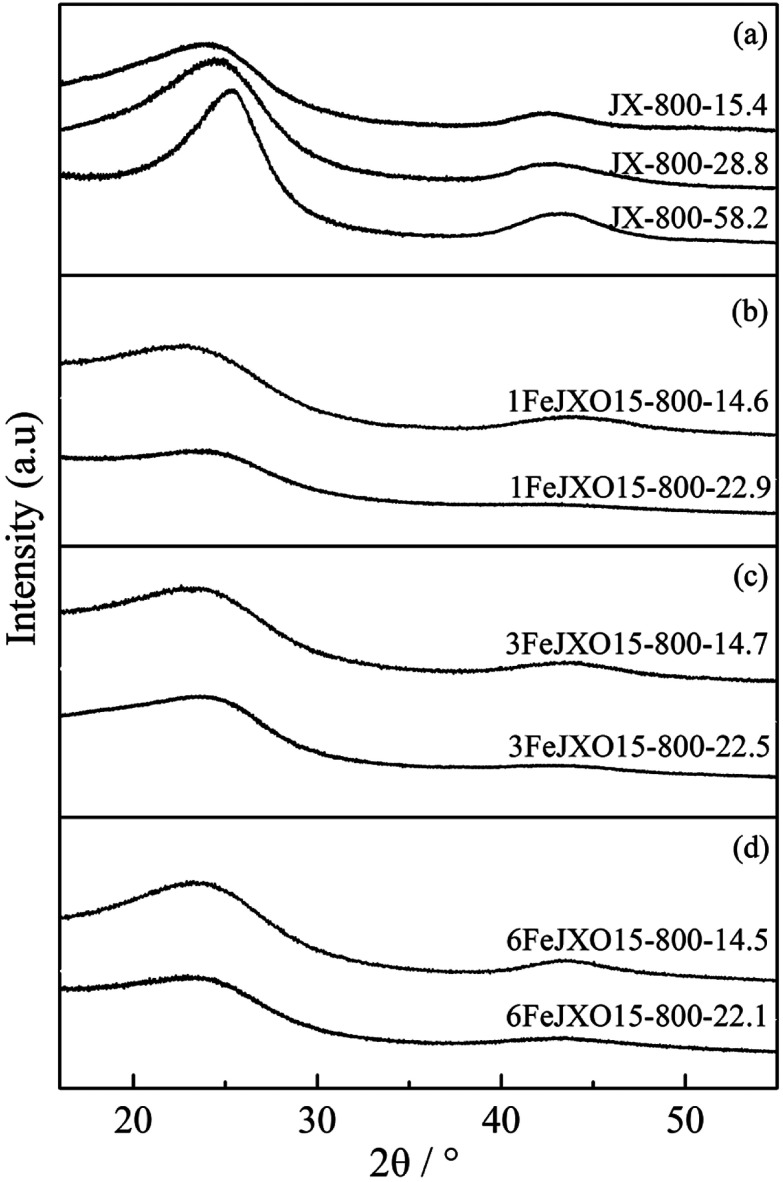
XRD profiles of coal chars at different burn offs during activation.

The XRD profiles and parameters of JX-800, 1FeJXO15-800H, 3FeJXO15-800H, and 6FeJXO15-800H at different burn offs during activation are shown in Fig. 5 and Table 4.

**Table 4:** XRD data of coal chars at different burn offs during activation

Samples	*L*_a_ (Å)	*L*_c_ (Å)	*d*_002_ (Å)	*N*
JX-800	24.91	13.72	3.50	3.77
JX-800-15.4	25.37	13.41	3.45	3.89
JX-800-28.8	26.78	13.92	3.36	4.14
JX-800-58.2	28.35	15.53	3.11	4.99
1FeJXO15-800H	25.71	12.95	3.69	3.51
1FeJXO15-800H-14.6	25.12	12.35	3.80	3.25
1FeJXO15-800H-22.9	22.67	10.91	4.32	2.53
3FeJXO15-800H	25.55	12.86	3.72	3.50
3FeJXO15-800H-14.7	24.88	12.19	3.90	3.13
3FeJXO15-800H-22.5	22.21	10.42	4.55	2.29
6FeJXO15-800H	25.13	12.43	3.76	3.31
6FeJXO15-800H-14.5	24.91	12.21	3.84	3.18
6FeJXO15-800H-22.1	23.76	11.20	4.16	2.69

There was a sustained increase in the *L*_a_ value and decrease in the *d*_002_ value for JX-800, and the *L*_c_ value first decreased at low burn offs from 0 to 15.4% and then increased from 15.4 to 58.2%, as shown in Table 4. It can be concluded that the microcrystalline structure has transformed into a highly ordered structure during activation. At the beginning of activation, the defects and some sandwich materials (such as aliphatic side chain and amorphous carbon) of the longitudinal aromatic layer were removed gradually; this resulted in a decrease in the *d*_002_ and *L*_c_ values. With an increase in carbon loss, the longitudinal aromatic layers began to condense and distort; this increased the thickness of the microcrystalline structure. The sustained increase of the *L*_a_ value was related to the rapid condensation of transverse aromatic layers because of the ordered orientation of aromatic layers produced by pyrolysis.

Alternatively, there was a sustained increase in the *d*_002_ value and a persistent decrease in the *L*_a_ and *L*_c_ values for 1FeJXO15-800H, 3FeJXO15-800H, and 6FeJXO15-800H with an increase in burn offs. At the beginning of activation, oxygen-containing active sites were removed gradually by active gas; thus, the addition of FeCl_3_ catalyst had promoted the disordered conversion of the microcrystalline structure during activation. In the catalytic process, the longitudinal aromatic structure condensed and distorted, and this occurred simultaneously with catalytic cracking; this resulted in an obvious decrease in the *L*_c_ value. Iron atom might have been bonded and fixed to carbon matrix, which destroyed the parallelism of the layer and the constancy of the interlayer spacing, thus increasing the interlayer spacing (*d*_002_). Fe-based compounds accelerate the etching of crystallite and always hinder the condensation and growth of crystallite simultaneously. However, the changed degree of XRD parameter for 6FeJXO15-800H decreased gradually at the high burn-off values ranging from 14.5 to 23.76%. The aggregation of the FeCl_3_ catalyst inside chars at high activation temperatures weakens the catalytic capacity, strengthening the condensation of longitudinal and transverse aromatic layers.

### 3.6. Carbon structure analysis of typical chars during activation

**Fig. 6:**
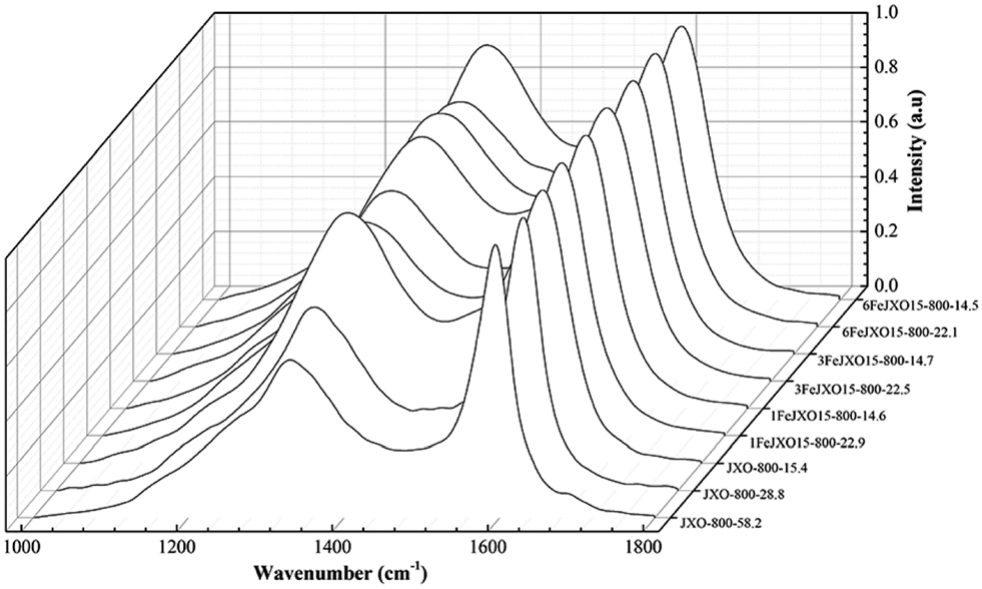
Raman spectra of coal chars at different burn offs during activation.

The Raman spectra and carbon structure parameters of JX-800, 1FeJXO15-800H, 3FeJXO15-800H, and 6FeJXO15-800H at different burn offs during activation are shown in Fig. 6 and Table 5.

**Table 5:** Raman data of coal chars at different burn offs during activation

Samples	*I*_D1_/*I*_G_	*I*_D3_/*I*_G_	*I*_D4_/*I*_G_	*I*_D1_/*I*_D3_
JX-800	3.197	1.922	0.611	1.663
JX-800-15.4	3.754	1.845	0.503	2.034
JX-800-28.8	4.514	1.797	0.314	2.511
JX-800-58.2	2.64	1.545	0.107	1.708
1FeJXO15-800H	3.770	2.715	0.849	1.388
1FeJXO15-800H-14.6	3.515	3.001	0.853	1.171
1FeJXO15-800H-22.9	3.089	3.450	0.864	0.895
3FeJXO15-800H	3.877	2.820	0.885	1.375
3FeJXO15-800H-14.7	3.573	3.175	0.890	1.125
3FeJXO15-800H-22.5	3.053	3.725	0.973	0.819
6FeJXO15-800H	3.987	2.955	0.936	1.349
6FeJXO15-800H-14.5	3.712	3.145	0.940	1.180
6FeJXO15-800H-22.1	3.499	3.513	0.951	0.996

There was a sustained decrease in the *A*_D3_/*A*_G_ and *A*_D4_/*A*_G_ values, whereas *A*_D1_/*A*_D3_ and *A*_D1_/*A*_G_ values first increased at the low burn offs ranging from 0 to 28.8% and then decreased from 28.8 to 58.2% for JX-800. At the beginning of activation, the smaller aromatic ring structure and active sites (such as cross-linking bonds) were consumed by activated gas preferentially. On the one hand, aromatic ring grew due to the dehydrogenation of hydro-aromatics during activation;^44^ on the other hand, the small aromatic ring structures might react with activated gas or convert into big aromatic ring structures at high activation temperatures;^46^ this resulted in a decrease in the *A*_D3_/*A*_G_ and *A*_D4_/*A*_G_ values and increase in the *A*_D1_/*A*_D3_ and *A*_D1_/*A*_G_ values. At the high burn-off from 28.8 to 58.2%, while the smaller aromatic ring structures were removed or changed preferentially, the inner aromatic structure could be activated by the continuous penetration of the activated gas; this could further induce the condensation of the aromatic ring and promote the formation of the crystalline sp^2^ structure.

Alternatively, there was a sustained decrease in the *A*_D1_/*A*_G_ and *A*_D1_/*A*_D3_ values and increase in the *A*_D3_/*A*_G_ value for 1FeJXO15-800H, 3FeJXO15-800H, and 6FeJXO15-800H, whereas a gentle increase in the *A*_D4_/*A*_G_ value was observed, as shown in Fig. 10. It could be inferred that the presence of FeCl_3_ catalyst could change the reaction pathways between the carbon structure and activated gas; the small aromatic ring systems and active sites could no longer be consumed with activated gas preferentially; the big aromatic rings would begin to decompose into small aromatic rings,^47,48^ and the FeCl_3_ catalyst could hinder the formation of the crystalline sp^2^ structure. In other words, the catalysts appeared to be preferentially accommodated on the carbons of aromatic nature, and more new cross-linking structures were formed from the broken fragments that resulted from the breakdown of aromatic structures by the catalytic capacity of FeCl_3_ catalyst. Moreover, the presence of O-containing structures was conducive to the reorganization of aromatic fragments. However, catalysts might move gradually and agglomerate with each other on the char surface at high burn-offs; this would lead to the degradation of catalysis for 6FeJXO15-800H.

### 3.7. Pore structure development of typical chars during activation

**Fig. 7:**
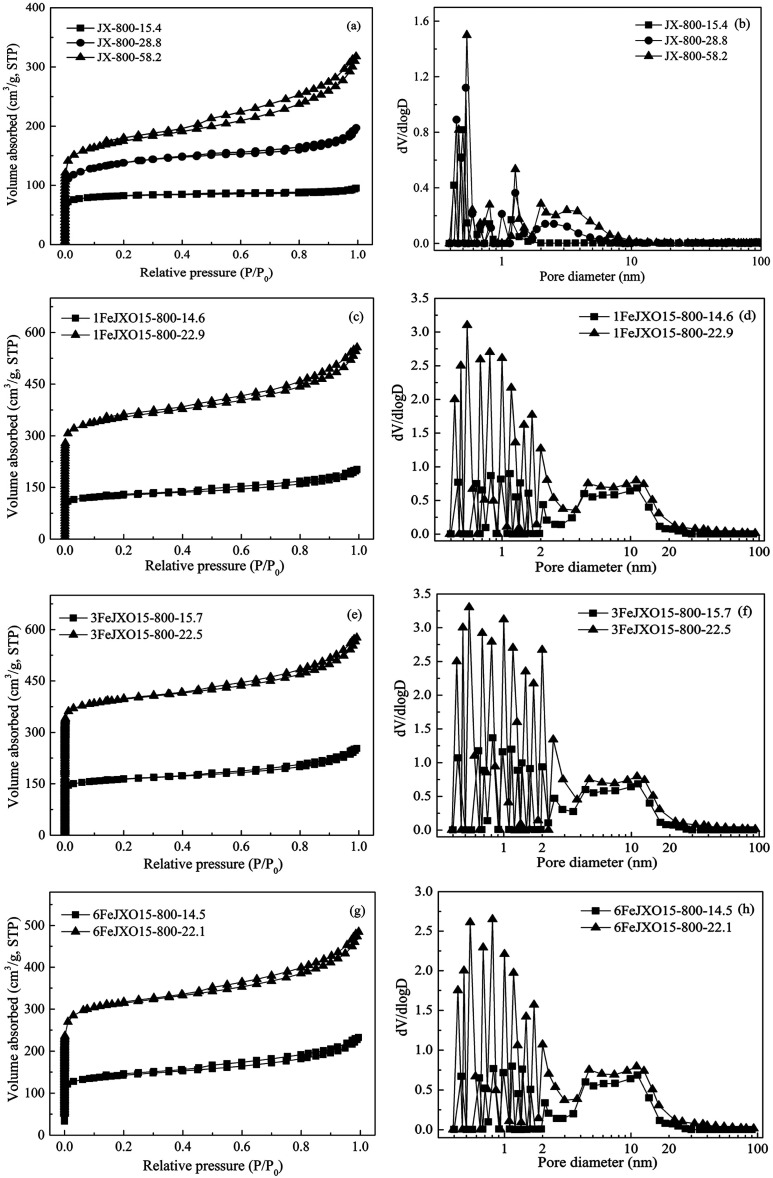
N_2_ adsorption isotherms and pore-size distributions of coal chars at different burn offs during activation.

To analyze the pore development of JX-800, 1FeJXO15-800H, 3FeJXO15-800H, and 6FeJXO15-800H at different burn-off values during activation, the N_2_ adsorption isotherms, the pore-size distribution, and parameters of porous structure are shown in Fig. 7 and Table 6.

**Table 6:** Pore structure parameters of typical chars at different burn offs during activation

Samples	*S*_BET_ (m^2^ g^−1^)	*V*_t_ (m^3^ g^−1^)	*V*_mic_ (m^3^ g^−1^)	Non-*V*_mic_ (%)	*D*_ap_ (nm)
JX-800-15.4	107.56	0.063	0.060	4.76	2.34
JX-800-28.8	305.45	0.154	0.127	17.53	2.02
JX-800-58.2	564.19	0.275	0.185	32.72	1.95
1FeJXO15-800H-14.6	348.45	0.192	0.140	27.08	2.20
1FeJXO15-800H-22.9	1045.43	0.344	0.285	17.15	1.32
3FeJXO15-800H-14.7	398.78	0.215	0.169	21.39	2.15
3FeJXO15-800H-22.5	1274.64	0.418	0.366	12.44	1.31
6FeJXO15-800H-14.5	326.55	0.184	0.130	29.34	2.25
6FeJXO15-800H-22.1	916.23	0.325	0.268	17.54	1.42

**Fig. 8:**
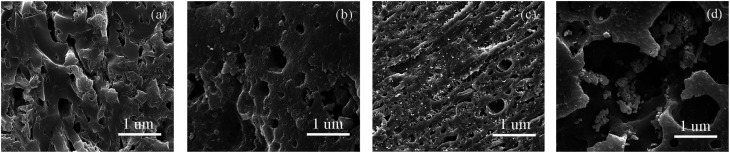
SEM images of coal chars under final burn-off values (a) 1FeJXO15-800H-22.9 (b) 3FeJXO15-800H-22.5 (c) 6FeJXO15-800H-22.1 (d) JX-800-58.2.

At first, based on Fig. 7, the isotherms of JX-800-15.4 could be classified as type I at low burn-offs, showing a narrow size distribution of less than 1 nm. With the gradual increase of burn-offs from 15.4% to 58.2%, all the N_2_ isotherms exhibited the characteristics of type I at low pressures as well as that of type IV at high pressures, and the clear hysteresis loops were observed, showing the formation of the hierarchical structure. Then, the pore structure parameters of JX-800 at different burn offs during activation are included in Table 6. The *S*_BET_ value of 107.56 m^2^ g^−1^, *V*_mic_ value of 0.06 m^3^ g^−1^, and non-*V*_mic_ value of 4.76% for JX-800-15.4 were obtained, indicating the major development of micropores during the initial activation process. There was a sustained decrease in the *S*_BET_, *V*_mic_, and non-*V*_mic_ values with an increase in the burn-off from 15.4% to 58.2%. This increase could be associated with the enlargement of micropores into mesopores progressively and the formation of many new micropores during this stage of activation. However, compared to the development of new micropores, the rapid increase of non-*V*_mic_ value, namely, the increase and development of mesopore, was more obvious from about 28.8% burn-off. Finally, the *S*_BET_ value of 564.19 m^2^ g^−1^ for JX-800-58.2 at relatively high burn offs was obtained, indicating a low *S*_BET_/burn-off ratio value of 9.69 m^2^ g^−1^/%, and severe carbon losses on the particle surfaces could also be observed, as shown in Fig. 8(d). These results indicated that the pore formation of JX-800 with less initial pores followed the hierarchical development from the surface to the core during activation. Moreover, the ordered conversion of microstructure hindered the penetration of activated gas into the interior of char structure; this led to the occurrence of more reactions on the particle surfaces rather than in the interior to decrease the production of the pores.

Alternatively, the adsorption capacities of 1FeJXO15-800H, 3FeJXO15-800H, and 6FeJXO15-800H increased rapidly at low pressures, but had little changes at high pressures with an increase in the burn-off from 14.5% to 22.9%; this indicated the major development of micropores. The *S*_BET_ and *V*_mic_ values rapidly increased during the whole stage of activation, whereas the non-*V*_mic_ value decreased drastically with the increasing burn-off. These changes meant that the initial pores of 1FeJXO15-800H, 3FeJXO15-800H, and 6FeJXO15-800H acted as channels to help the diffusion of activated gas, and the oxygen-containing active sites could strengthen the etching of the carbon structure. Along with the gradual consumption of oxygen functional groups, the disordered conversion of carbon structure and more active sites in the presence of Fe-based compounds facilitated a sustained production of more micropores; this resulted in the rapid increase of the *S*_BET_, *V*_t_, and *V*_mic_ values. Although the catalysts might have moved and agglomerated on the particle surfaces for 6FeJXO15-800H with an increase of burn-off, no severe carbon losses occurred on the particle surface, as observed in Fig. 8(a)–(c). Importantly, the effects of oxygen functional groups and FeCl_3_ catalyst could further facilitate the rapid development of micropores at a relatively low burn off. The *S*_BET_ values (1045.43, 1274.64, and 916.23 m^2^ g^−1^) of 1FeJXO15-800H, 3FeJXO15-800H, and 6FeJXO15-800H at the relatively low burn-off values of 22.9%, 22.5%, and 22.1% were obtained, indicating the high values *S*_BET_/burn-off ratio of 45.65 m^2^ g^−1^/%, 56.65 m^2^ g^−1^/%, and 41.46 m^2^ g^−1^/%, respectively.

## 4 Conclusions

Physical activation of Jixi bituminous coal regulated by oxygen functional groups (created by air pre-oxidation at 200 °C for 15 h) and FeCl_3_ (1 wt%, 3 wt%, and 6 wt%) has provided a good control of micro- and macro-structure of Jixi bituminous char throughout the preparation process to obtain the ideal AC production with a high *S*_BET_ at low burn-off values. In the phase of pyrolysis, the existence of oxygen functional groups could improve the distributed formation of FeCl_3_ catalyst in the interior of the particle, hindering the concentration and amalgamation of FeCl_3_ catalyst at high temperatures and promoting the catalytic characteristics. The effects of oxygen functional groups and FeCl_3_ catalyst could further promote the disordered conversion of microstructure and the number of active sites, but catalytic cracking characteristics of the FeCl_3_ catalyst could play a more important role. In addition, FeCl_3_ could efficaciously promote the development of micropores, and the evolution of oxygen functional groups mainly facilitated the production of mesopores and macropores. In the phase of activation, the reaction pathway of active sites and activated gas was changed by the presence of the FeCl_3_ catalyst; this resulted in a consistent disordered conversion of the microstructure of 1FeJXO15-800H, 3FeJXO15-800H, and 6FeJXO15-800H as compared to the ordered conversion of JX-800. The effects of oxygen functional groups and FeCl_3_ catalyst could promote the favorable diffusion of activated gas throughout more initial pores produced by oxygen functional groups, following non-hierarchical development. With an increase in activation time, the existence of FeCl_3_ catalyst could facilitate the etching of the carbon structure; this resulted in a rapid and continuous development of the micropores rather than the severe carbon losses on the particle surface. Finally, the *S*_BET_ values (1045.43, 1274.64, and 916.23 m^2^ g^−1^) of 1FeJXO15-800H-22.9, 3FeJXO15-800H-22.5, and 6FeJXO15-800H-22.1 were obtained, indicating the high *S*_BET_/burn-off ratio values of 45.65 m^2^ g^−1^/%, 56.65 m^2^ g^−1^/%, and 41.46 m^2^ g^−1^/%, respectively, as compared to the relatively low *S*_BET_ 564.19 m^2^ g^−1^ of JX-800-58.2, indicating a low *S*_BET_/burn-off ratio value of 9.69 m^2^ g^−1^/%.

## Conflicts of interest

There are no conflicts of interest to declare.

## Supplementary Material

RA-008-C7RA12928A-s001
