# Supplementary material for: Effects of oxygen functional groups and FeCl3 on the evolution of physico-chemical structure in activated carbon obtained from Jixi bituminous coal
Source: RSC Adv. 2018 Feb 26;8(16):8569–79. doi: 10.1039/c7ra12928a (PMC9078562; doi:10.1039/c7ra12928a)
Supplement: RA-008-C7RA12928A-s001 [file RA-008-C7RA12928A-s001.pdf]

Electronic Supplementary Material (ESI) for RSC Advances.
This journal is © The Royal Society of Chemistry 2018

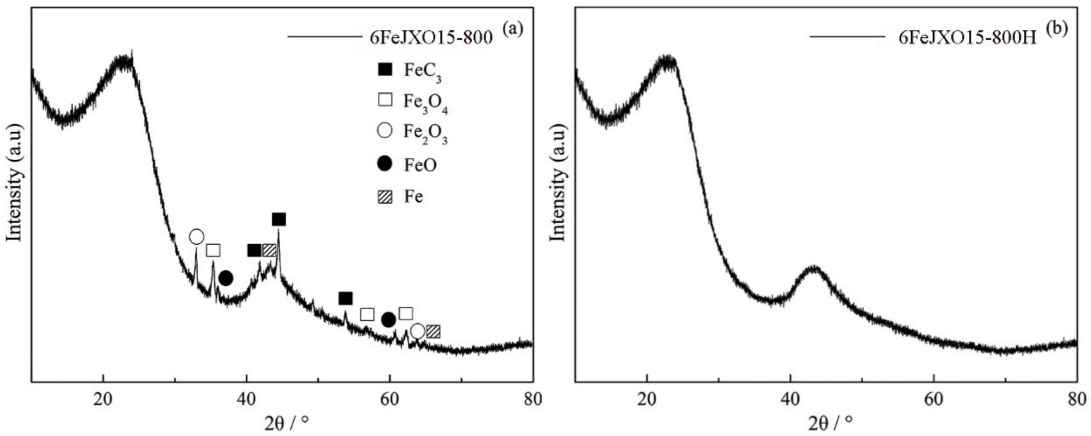

Fig.S1 XRD phase analysis of 6FeJXO15-800 and 6FeJXO15-800H

There are Fe-based components (such as Fe_3O_4, Fe_2O_3, FeO, Fe and Fe_3C) for 6FeJXO15-800, as shown in Fig. S1(a), indicating that $FeCl_3$ catalyst has reacted with some substances in coal during pyrolysis. To eliminate the interference of Fe-based compounds in chars for the results of XRD and Raman, some char samples including Fe-based compounds were treated by 0.2 mol L^{-1} HCl and washed with distilled water to remove chloride ions. After acid treatment, the diffraction peak of Fe-based components has disappeared in Fig. S1(b).
